# Glycogen Synthase Kinase 3β Inhibition as a Therapeutic Approach in the Treatment of Endometrial Cancer

**DOI:** 10.3390/ijms140816617

**Published:** 2013-08-12

**Authors:** Yan Yin, Nora T. Kizer, Premal H. Thaker, Katherine B. Chiappinelli, Kathryn M. Trinkaus, Paul J. Goodfellow, Liang Ma

**Affiliations:** 1 Division of Dermatology, Department of Medicine, Washington University School of Medicine, 660 South Euclid Avenue, MO 63110, USA; E-Mail: yyin@dom.wustl.edu; 2 Department of Obstetrics and Gynecology, Washington University School of Medicine, 660 South Euclid Avenue, MO 63110, USA; E-Mails: kizern@wudosis.wustl.edu (N.T.K.); thakerp@wustl.edu (P.H.T.); paul.goodfellow@osumc.edu (P.J.G.); 3 Division of Endocrine and Oncologic Surgery, Department of Surgery, Washington University School of Medicine, 660 South Euclid Avenue, MO 63110, USA; E-Mail: kate.chiappinelli@gmail.com; 4 Division of Biostatistics, Washington University School of Medicine, 660 South Euclid Avenue, MO 63110, USA; E-Mail: kimt@wubios.wustl.edu; 5 Department of Developmental Biology, Washington University School of Medicine, 660 South Euclid Avenue, MO 63110, USA

**Keywords:** lithium chloride, GSK3β, endometrial cancer

## Abstract

Alternative strategies beyond current chemotherapy and radiation therapy regimens are needed in the treatment of advanced stage and recurrent endometrial cancers. There is considerable promise for biologic agents targeting the extracellular signal-regulated kinase (ERK) pathway for treatment of these cancers. Many downstream substrates of the ERK signaling pathway, such as glycogen synthase kinase 3β (GSK3β), and their roles in endometrial carcinogenesis have not yet been investigated. In this study, we tested the importance of GSK3β inhibition in endometrial cancer cell lines and *in vivo* models. Inhibition of GSK3β by either lithium chloride (LiCl) or specific GSK3β inhibitor VIII showed cytostatic and cytotoxic effects on multiple endometrial cancer cell lines, with little effect on the immortalized normal endometrial cell line. Flow cytometry and immunofluorescence revealed a G2/M cell cycle arrest in both type I (AN3CA, KLE, and RL952) and type II (ARK1) endometrial cancer cell lines. In addition, LiCl pre-treatment sensitized AN3CA cells to the chemotherapy agent paclitaxel. Administration of LiCl to AN3CA tumor-bearing mice resulted in partial or complete regression of some tumors. Thus, GSK3β activity is associated with endometrial cancer tumorigenesis and its pharmacologic inhibition reduces cell proliferation and tumor growth.

## 1. Introduction

Endometrial carcinoma is the fourth most common cancer and the most common invasive gynecological malignancy among U.S. women, with an estimated 47,130 new cases and 8010 deaths in 2012 [1]. Despite advancements in therapy over the last 20 years, both incidence and five-year survival rates have remained roughly unchanged. Endometrial carcinoma is commonly classified into two pathogenetic groups: type I being less invasive and low-grade with generally good prognosis, and type II being unassociated with exposure to estrogen and often high-grade and more invasive. Standard chemotherapy for metastatic endometrial carcinoma consists of treatments with cytotoxic agents including anthracyclines, platinum compounds and taxanes. Response rates of single agent therapy range from 12% to 42%, and those of combinations of cytotoxic chemotherapy range from 33% to 57% [2]. Alternative treatment options beyond chemotherapy and radiation therapy are currently limited. Several biologic agents have been or are currently being investigated, but there is still a clear need for novel single agent or combination therapies for metastatic and recurrent endometrial cancer.

Biological therapies that target the extracellular signal-regulated (ERK) pathway for the treatment of endometrial cancer represent a particularly promising avenue of investigation. ERK activation occurs in greater than 50% of endometrial cancers, as evidenced by phospho-ERK positivity measured by immunohistochemistry ([3] and our unpublished findings). Approximately 15% of endometrial tumors carry fibroblast growth factor receptor (FGFR) 2 mutations and a similar fraction have KRAS mutations [4–6]. The fact that FGFR2 and KRAS mutations are mutually exclusive in endometrial cancers points to the importance of activation of the ERK pathway [5]. Additionally, ERK activation is seen in endometrial cancers without mutations in KRAS and FGFR2 [3] (and our unpublished data). At present the role that the ERK signaling pathway plays in both the normal endometrium and endometrial tumorigenesis is poorly understood. Furthermore, the downstream substrates of ERK signaling that are important in the endometrial epithelium and their roles in endometrial carcinogenesis remain largely unknown.

Loss-of-function (LOF) mutations of PTEN, a tumor suppressor gene, are frequently found in endometrial cancers [7,8]. PTEN, a lipid phosphatase, functions as a negative regulator of the phosphatidylinositol 3-kinase (PI3K) signaling cascade [9]. PTEN LOF mutations thus activate the PI3K pathway, resulting in phosphorylation and activation of the key component protein kinase B (PKB, aka AKT), and elicit a survival signal that promotes cell proliferation and protects cells from undergoing apoptosis induced by various stresses [10,11]. Other alterations that could result in PI3K/AKT pathway activity changes are mutations in the PIK3CA (p110α) gene, which encodes the catalytic subunit of PI3K, as well as the PIK3R1 (p85α) and PIK3R2 (p85β) genes, which both encode inhibitory subunits of PI3K. Somatic mutations of these genes were frequently detected in primary endometrial cancers, most of which resulted in gain of function of the PI3K pathway [12–14]. In addition, these mutations are often accompanied by PTEN mutations, and are thought to have an additive effect to PTEN monoallelic inactivation in endometrial carcinogenesis [15]. PKB/AKT exerts its functions through phosphorylation of substrates that are involved in the crucial steps of cell survival and cell cycle progression, including the forkhead box transcription factors (FOXO), the cell-cycle inhibitor p27, the mTOR pathway inhibitor tuberous sclerosis complex 2 (TSC2), and glycogen synthase kinase 3 (GSK3) [16–19]. GSK3β, a serine/threonine kinase involved in many fundamental cellular processes, is a particularly important downstream mediator of the AKT/PI3K pathway as it regulates a variety of carcinogenic events including cell survival, proliferation and differentiation [20–22]. It has been shown that GSK3β can be phosphorylated by the ERK kinase, and can modulate the activity of several major tumorigenic pathways including the canonical WNT pathway, NF-κB signaling, and the p53-induced apoptotic pathway. The most established role for GSK3β is to phosphorylate β-catenin, the critical mediator of canonical WNT signaling, which results in its degradation. Notably, the role for GSK3β in carcinogenesis is largely context dependent. In certain cancers, GSK3β functions as a tumor suppressor gene and its inactivation is associated with Cdc25a overexpression [23]. In contrast, accumulating evidence suggest that GSK3β functions as an oncogene and promotes proliferation and augments cell survival, and has been proposed as a potential therapeutic target for cancer treatment [24]. Treatment with LiCl, a chemical inhibitor of GSK3β, promotes survival of mice in a leukemia model [25]. Similarly, LiCl treatment selectively decreased cell proliferation in breast and colon cancer cells with a mutant PIK3CA knock-in [26].

The function of GSK3β in endometrial carcinogenesis has not been fully elucidated. In the current study, we explored effects of GSK3β inhibitors, lithium chloride and GSK3β inhibitor VIII (AR-A014418), on multiple human endometrial cancer cell lines. Both reagents inhibited endometrial cancer cell growth by inducing cell cycle arrest. Treatment of mice carrying human endometrial cancer xenografts with lithium-supplemented water showed evidence of complete or partial regression in some tumors. Our results indicate that GSK3β activity is associated with endometrial cancer tumorigenesis and that its pharmacologic inhibition reduces cell proliferation and tumor growth.

## 2. Results

### 2.1. Cytostatic Effect of Lithium Chloride Treatment in Endometrial Cancer Cell Lines

**Figure 1:**
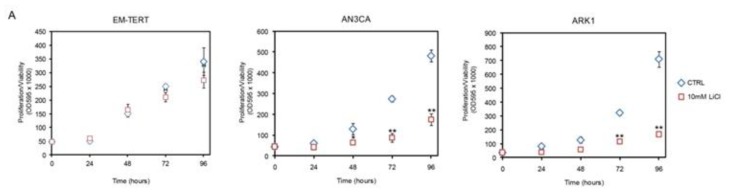

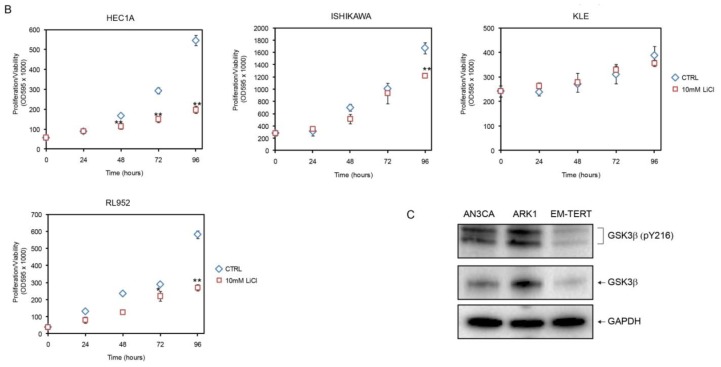
Growth inhibitory effects of LiCl on endometrial cancer cell lines. (**A**,**B**) Representative results for MTT assays performed 0–96 h post treatment with 10 mM LiCl. Cell proliferation/viability was significantly reduced in five of six endometrial cancer cell lines, with no effects seen with the immortalized human endometrial cell line, EM-TERT or the KLE tumor cell line. *p < 0.05; **p < 0.01 compared to the control by Student’s *t*-test; (**C**) Western blot revealed high level of active GSK3β in AN3CA and ARK1 cancer cell lines.

To test the effect of GSK3β inhibition on endometrial cancer cell growth, the endometrial cancer cell lines AN3CA (type I) and ARK1 (type II), as well as an immortalized normal endometrial epithelial cell line EM-E6/E7/TERT (hereinafter referred to as EM-TERT) [27] were treated with control media or media with 10 mM LiCl. The effect of LiCl treatment was assessed using the MTT assay at 24-hour intervals out to 96 h. LiCl treatment resulted in reduced cell viability/proliferation in both the AN3CA and ARK1 cancer cell lines in a time-dependent manner, whereas EM-TERT control cells were not affected (Figure 1). Treatment of the AN3CA with 10 mM LiCl resulted in a significant reduction in cell viability/proliferation by 48 h (50.3% reduction, *p* = 0.04), and the reduction remained significant through 72 and 96 h (69.1% reduction, *p* < 0.0005 and 63.7% reduction, *p* < 0.0005, respectively compared to controls,). Similar effects on ARK1 cells were observed (54.6% reduction, *p* < 0.005 at 72 h and 76.2% reduction, *p* < 0.005 at 96 h, respectively compared to controls). In both AN3CA and ARK1 cells, treatment of LiCl at a lower concentration of 1 mM showed reduced viability/proliferation; however, these effects varied between replicate experiments and seemed to be affected by seeding density (data not shown). No effect with treatment of 1 mM LiCl was observed in the EM-TERT cell line. Given that treatment with 10 mM LiCl showed an early, consistent and marked cytostatic effect in both cancer cell lines initially studied (AN3CA and ARK1), we used this dose to assess the effect of LiCl on growth of four additional endometrial cancer cell lines. Three out of the four cell lines (HEC1A, ISHIKAWA and RL952) exhibited reduced cell viability/proliferation at 96 h, with HEC1A and RL952 cells showing an early response. KLE cells, like the EM-TERT normal endometrial epithelial cell line, showed no change in growth as measured by MTT over the time course tested. The KLE cells have a noticeable longer doubling time than the other cell lines investigated (Figure 1, note the reduced steepness of the growth curve of KLE compared to all other cell lines), hence it is possible that the increased length of cell cycle over the 96 h treatment period may have masked any effect of LiCl on its growth. Western blot analysis confirmed the inhibitory effect of LiCl on GSK3β, as LiCl treatment resulted in increased levels of phosphorylation of serine 9 residue (pSer9) GSK3β [28,29] in both the EM-TERT and AN3CA cell lines, with AN3CA cancer cells showing a higher pSer9/total GSK3β ratio (Figure S1). Intriguingly, Western Blot on active form of GSK3β (pTyr216) revealed a marked higher basal level in untreated AN3CA and ARK1 cell lines than the control EM-TERT cell line (Figure 1C), indicating abnormal hyperactivity of GSK3β in endometrial cancer cell lines.

**Figure 2:**
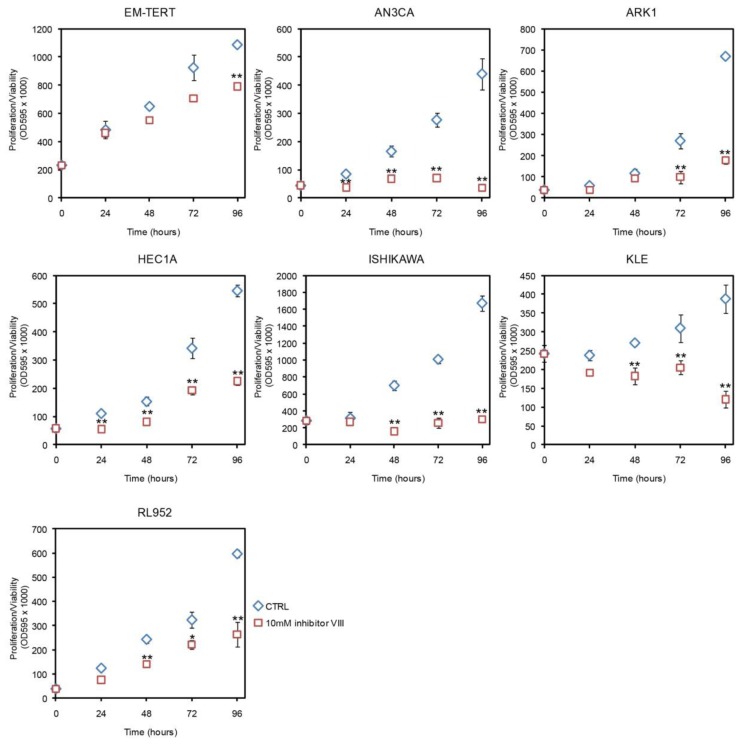
Effects of GSK3β inhibitor VIII measured by MTT. GSK3β inhibitor VIII had a modest effect on EM-TERT cells, whereas cell proliferation/viability was substantially reduced in cancer cell lines. *p < 0.05; **p < 0.01.

To determine whether the LiCl effect seen in the endometrial cancer cell lines was specific to GSK3 inhibition, we tested another GSK3β inhibitor, GSK3β inhibitor VIII [30], and found similar results (Figure 2). At 10 μM, GSK3β inhibitor VIII inhibited cell growth of all six endometrial cancer cell lines tested, while only modestly affecting EM-TERT cells. The growth inhibitory effect was observed in all five endometrial cancer cell lines that had shown LiCl inhibition, with significant reductions in proliferation/viability as early as 24 h post treatment (Figure 2). KLE, the single endometrial cancer cell line that did not respond to 10 mM LiCl treatment, showed significant growth reduction in response to inhibitor VIII.

To determine if the effects seen in the MTT assays reflected cytostatic or cytotoxic effects of each of the agents tested (or combinations of cytostatic and cytotoxic effects), we performed trypan blue exclusion assays in a subset of cell lines. LiCl treatment at 10 mM did not result in cell death in EM-TERT (2.91% ± 1.62% *vs.* 2.39% ± 1.10%); however, cell death was increased both in AN3CA cells (15.0% ± 7.5% *vs.* 3.1% ± 1.4%) and in ARK1 cells (4.4% ± 0.6% *vs.* 2.2% ± 0.2%). GSK3β inhibitor VIII, on the other hand, exhibited modest cell toxicity on EM-TERT cells at 10 μM (7.7% ± 3.6% *vs.* 2.4% ± 1.1%), but caused significant cell death in both cancer cell lines (66.5% ± 2.9% *vs.* 3.1% ± 1.4% in AN3CA; 14.4% ± 3.1% *vs.* 2.2% ± 0.2% in ARK1, Figure S2).

Taken together, these data demonstrate that reduced GSK3β activity inhibits endometrial cancer cell growth through both cytostatic and cytotoxic mechanisms, while minimally affecting that of the normal endometrial epithelial cells.

### 2.2. GSK3β Inhibition Induces G2/M Arrest in Endometrial Cancer Cell Lines

As LiCl-induced GSK3β inhibition reduced cell proliferation, we next evaluated the mechanism behind this cytostatic effect. GSK3 plays a key role in several pathways that target the cell cycle [23,31,32]. Specifically, GSK3β regulates cyclin D1 turnover and subcellular localization by catalyzing its phosphorylation on Thr-286 [33]. GSK3β is also a key player in the PKB/AKT pathway, which targets both cyclin D1 and p53 [32,34]. Thus, we sought to investigate whether the cytostatic effects we observed with inhibition of GSK3β were due to perturbation of the cell cycle.

**Figure 3:**
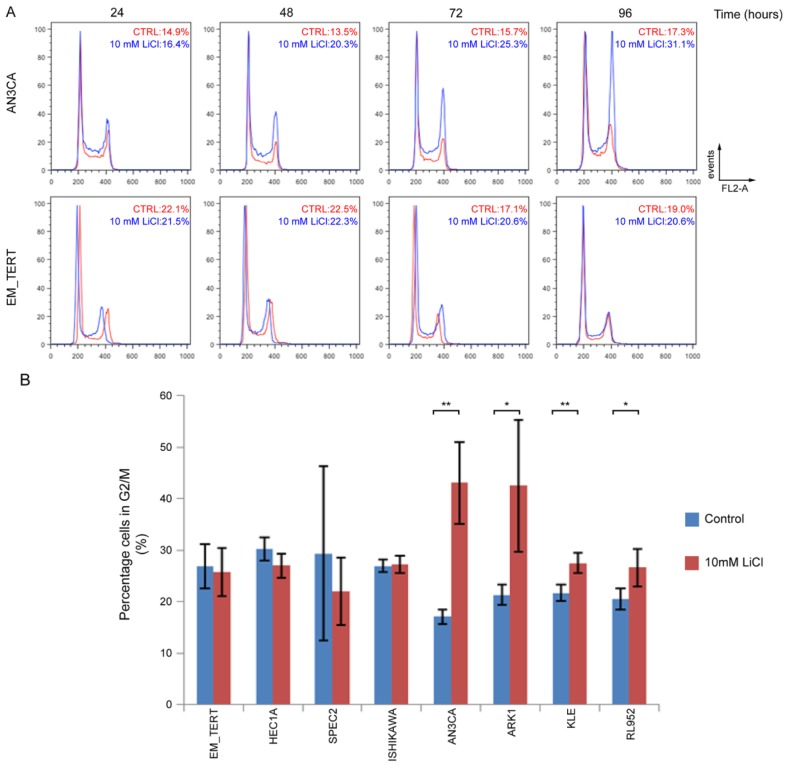
Endometrial cancer cells treated with LiCl accumulate at G2/M phase. (**A**) Representative FACS analyses on cell cycle progression in untreated (red) and 10 mM LiCl-treated (blue) endometrial cell lines. In AN3CA cells, percentage of cells in G2/M phase steadily increased and peaked at 96 h. No such changes were detected in the EM-TERT cell line. Percentages of cells in G2/M are indicated; (**B**) Quantification of G2/M cell percentages at 96 h comparing 10 mM LiCl-treated *vs.* control cells in multiple endometrial cell lines. At least three independent experiments were performed for each cell line. * denotes *p* < 0.05 and ** denotes *p* < 0.01.

Flow cytometry (FACS) to evaluate cell cycle progression revealed that treatment with 10 mM LiCl caused an accumulation of cells in G2/M phase in the endometrial cancer cell line AN3CA, but not in immortalized control cells, EM-TERT (Figure 3A). Accumulation was seen as early as 48 h after LiCl exposure and was sustained through 96 h of treatment. Further cell cycle analyses showed a similar accumulation of cells in G2/M phase in 4 of 7 endometrial cancer cell lines treated with 10 mM LiCl for 96 h (Figure 3B). A G2/M cell cycle arrest was observed in both type I (AN3CA, KLE, and RL952) and type II (ARK1) endometrial cancer cell lines. AN3CA cells treated with inhibitor VIII for 96 h showed a similar accumulation in G2/M phase (Figure S3). No cell cycle effects were observed for EM-TERT cells. In conclusion, decreased cell proliferation by GSK3β inhibition is likely mediated, at least in part, by inducing a G2/M cell cycle arrest.

### 2.3. GSK3β Inhibition-Induced Cancer Cell Arrest Is Cell Context-Dependent

**Figure 4:**
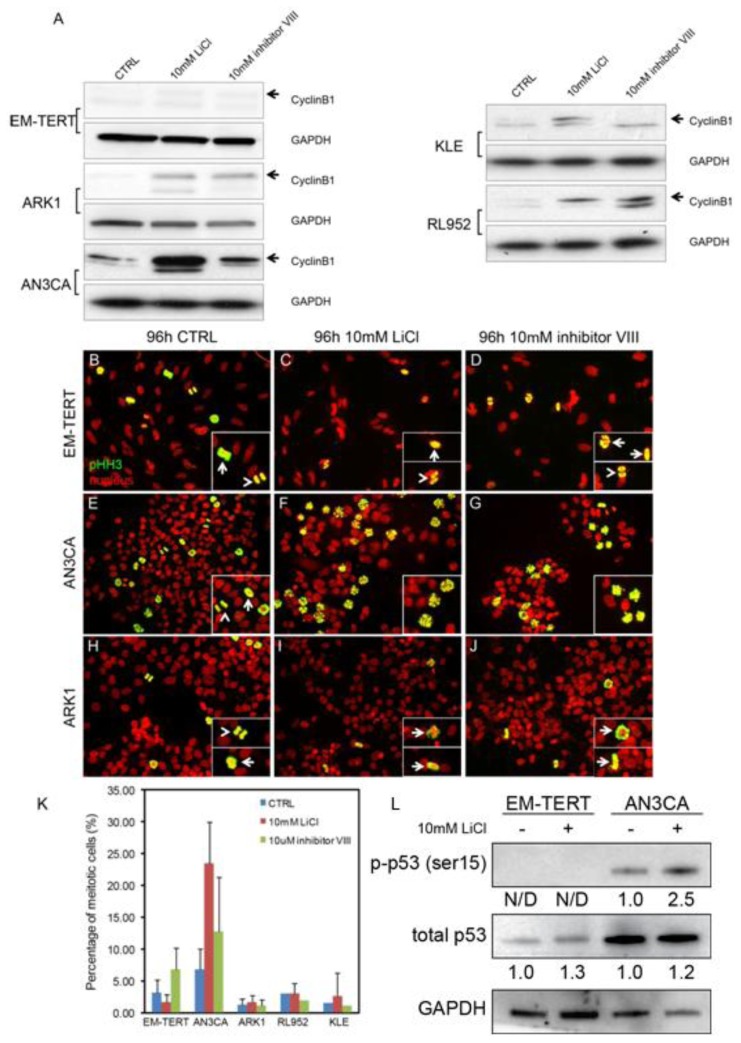
GSK3β inhibition-induced G2/M arrest is cell context-dependent. (**A**) Western blot on Cyclin B1 showed accumulation of this protein by either LiCl or GSK3β inhibitor VIII treatment for 96 h in multiple endometrial cancer cell lines without affecting the EM-TERT control cells; (**B**–**J**) Immunofluorescence of phospho-histone H3 (pHH3) showed accumulation of cells with staining pattern resembling that of the prophase cells in both LiCl- and GSK3β inhibitor VIII-treated AN3CA cells (F, G, insets). Note that normal mitotic cells at pro-/meta-phase (arrows) and ana-/telo-phase (arrowheads) in EM-TERT and ARK1 cells regardless of treatment (**B**–**D**, **H**–**J**, respectively) as well as untreated AN3CA cells (**E**); (**K**) Quantification of pHH3-positive cells showed an increase in mitotic cell percentage only in AN3CA cells after anti-GSK3β treatments; (**L**) Western Blot showed that LiCl induced p53 activation (pSer15) only in the AN3CA cells, but not in the EM-TERT cells. Total p53 level was not significantly affected by LiCl treatment in either cell line. Fold-change of normalized p-p53 and p53 level comparing to untreated control is indicated. N/D: not detected.

Our FACS analyses showed a G2/M arrest in endometrial cancer cells treated with GSK3β inhibitors, which prompted us to examine the expression of Cyclin B1, a cell cycle regulator that is rapidly up-regulated at G2 and degraded at mid-M-phase [35]. Consistent with the cell cycle arrest phenotype, elevated Cyclin B1 protein level was evident by Western blotting in ARK1, AN3CA and RL952 cancer cell lines treated with either LiCl or inhibitor VIII, whereas no induction of Cyclin B1 was seen in EM-TERT cells (Figure 4A).

To further elucidate at which stage of G2/M the endometrial cancer cells arrest after GSK3β inhibition, we performed immunofluorescence against phospho-histone H3 (pHH3), a well-established mitotic marker. In control EM-TERT cells, cells at different phases during mitosis were evident (Figure 3B, arrow points to pro-/meta-phase cells, arrowhead points to ana-/telo-phase cells). In EM-TERT cells treated with either 10 mM LiCl or 10 μM inhibitor VIII, mitotic cells at all stages were detected (Figure 4C,D) and the percentage of pHH3-positive cells was not changed significantly (Figure 4K, 3.18% ± 1.98% in control, 1.65% ± 1.21% after 10 mM LiCl treatment and 6.81% ± 3.39% after inhibitor VIII treatment). Mitotic cells at all stages were detected in control AN3CA (Figure 4E,K, 6.81% ± 3.20%); however, GSK3β inhibition with either agent caused evident pro-M phase arrest (Figure 3F,G, insets), and resulted in drastically increased numbers of pHH3-positive cells (Figure 4K, 23.47% ± 6.45% after LiCl treatment, *p* = 6.49 × 10^−9^; 12.73% ± 8.61% after inhibitor VIII treatment, *p* = 0.017).

Unlike AN3CA cells, ARK1 cells did not exhibit much change in pHH3 cell staining patterns or show an accumulation of pHH3-positive mitotic cells when treated with either LiCl or inhibitor VIII (Figure 4H–J, K, 1.24% ± 0.90% in control, 1.65% ± 1.09% after LiCl treatment, *p* = 0.25; 1.12% ± 0.94% after inhibitor VIII treatment, *p* = 0.22). The other two cell lines that showed a G2/M arrest with LiCl treatment, KLE and RL952, had pHH3 staining patterns similar to ARK1, making AN3CA the sole example of pro-M phase arrest (Figure 4J). ARK1, KLE, and RL952 cells all showed elevated Cyclin B1 levels concomitant with treatment, consistent with arrest at G2 phase as a result of GSK3β inhibition. Intriguingly, we observed activation of p53 (p-Serine 15), the tumor suppressor gene, only in the AN3CA endometrial cancer cells, but not in the normal EM-TERT cells (Figure 4L). It is well known that aberrant activation of p53 by DNA damage, or other genomic aberrations, could cause subsequent cell cycle arrest and/or apoptosis [36]. We did not, however, observe such a change in the type II ARK1 cancer cells (data not shown), suggesting that lithium-induced p53 activation is associated with pro-M phase cell cycle arrest in AN3CA endometrial cancer cells only. Altogether, these data demonstrated that GSK3β inhibition caused endometrial cancer cell cycle arrest at different stages during G2/M phase, possibly due to varying genetic make-up and cell context differences.

### 2.4. Genetic Knockdown of GSK3β Reduced Cell Viability/Proliferation in AN3CA Cells

**Figure 5:**
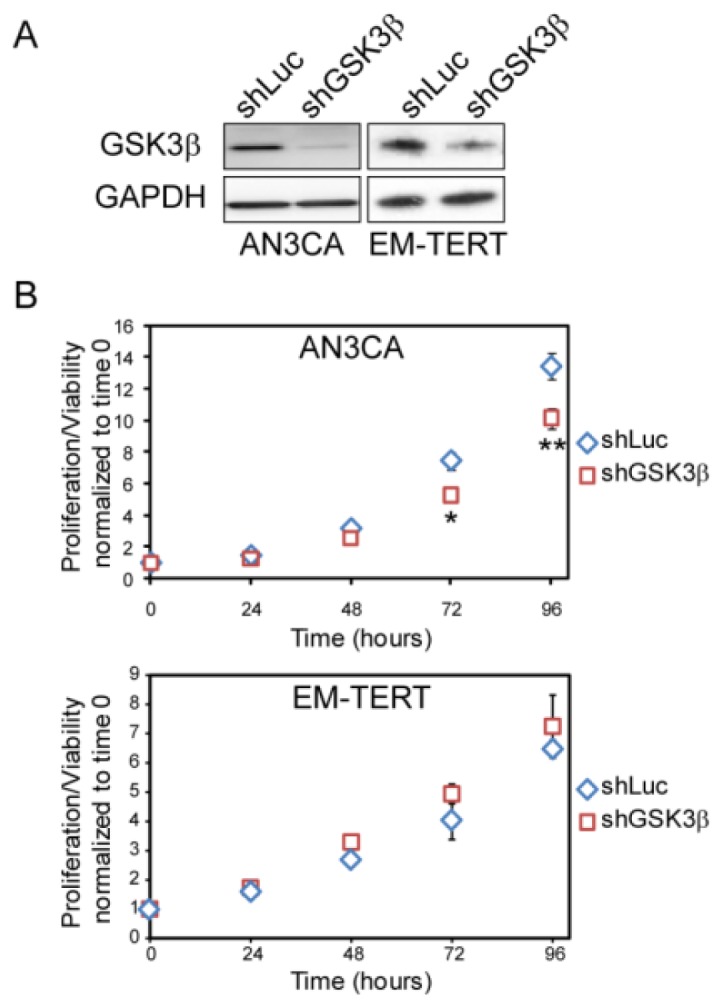
GSK3β knock-down by lentiviral shRNA showed similar effect on cell growth as compared to treatment with GSK3β inhibitors. Two shGSK3β knockdowns yielded similar results and data from shGSK3β-1 were shown. (**A**) Western blot of GSK3β demonstrating effective knock-down in AN3CA and EM-TERT cells by lentiviral infection; (**B**) MTT assay revealed that GSK3β knock-down inhibited AN3CA endometrial cancer cell growth, but had no effect on control EM-TERT cell growth. *p < 0.05; **p < 0.01 compared to the control by Student’s *t*-test.

To rule out any off-target effects by GSK3β inhibitors, we directly knocked down endogenous GSK3β by infecting AN3CA cancer cells and EM-TERT control cells with lentivirus carrying shRNA against human GSK3β. Two independent shRNA constructs were tested which yielded similar results. Reduction in GSK3β protein level was validated by western blotting as shown in Figure 5A. shRNA constructs significantly reduced total GSK3β level in both shGSK3β infected cells, compared to shLUC-infected cells. MTT assays showed that GSK3β knockdown reduced cell viability in the AN3CA cells (Figure 5B). At 72 and 96 h, GSK3β knock-down AN3CA cells showed significant reduced cell proliferation/viability compared to cells infected with shLUC construct. However, no effects of GSK3β knock-down were seen in the normal EM-TERT cell line. These results definitively confirmed the cytostatic effects of GSK3β inactivation in endometrial cancer cell lines.

### 2.5. GSK3β Inhibition Sensitizes Endometrial Cancer Cells to Paclitaxel

**Figure 6:**
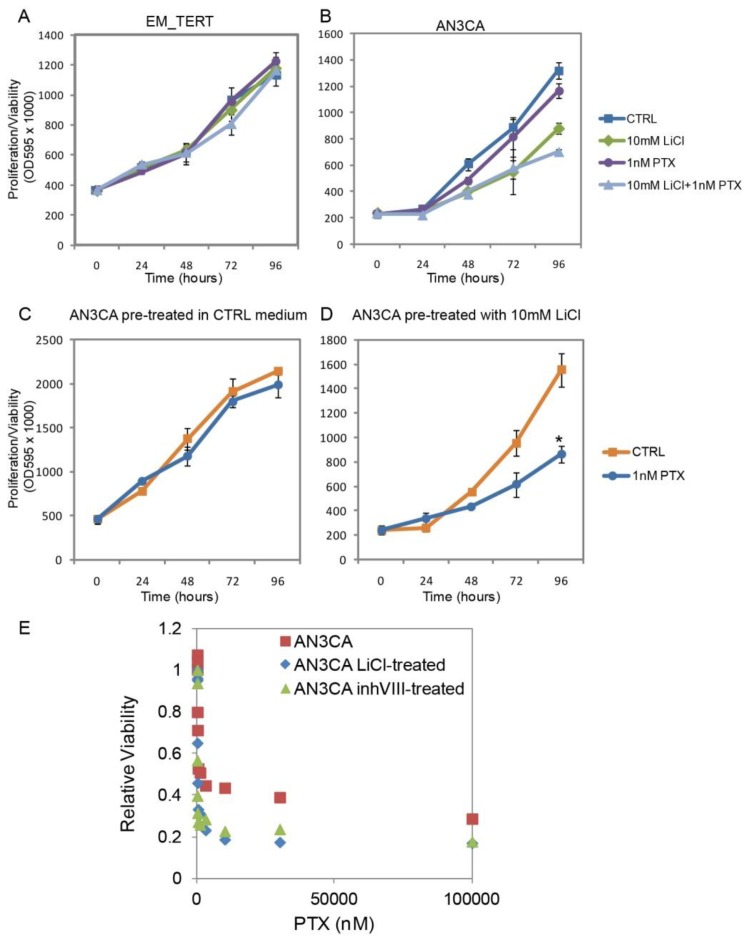
GSK3β inhibition sensitizes endometrial cancer cells to Paclitaxel (PTX). (**A**,**B**) Treatments with LiCl, PTX or the combination of the two agents did not interfere with EM-TERT cell growth, whereas combo-treatment showed a greater growth inhibition on AN3CA cells compared with either single agent treatment; (**C**,**D**) AN3CA cell exposed to LiCl for 96 h prior to PTX treatment showed a dramatically improved response to this drug; (**E**) AN3CA cells previously exposed to control, 10 mM LiCl or μM GSK3β inhibitor VIII were treated for 72 h with various concentrations of PTX. Cell survival was determined by MTT assays and calculated as the ratio of viable cells with respect to non-PTX-treated controls. IC_50_ values were determined by using the ED50plus online software [43].

Deregulation and signal imbalance secondary to GSK3β inhibition in cancer cells may make them more susceptible to chemotherapeutic agents. To test this hypothesis, we treated AN3CA and EM-TERT cells with a combination of LiCl and paclitaxel (PTX), a mitotic inhibitor widely prescribed to endometrial cancer patients. At 1 nM concentration, PTX alone did not affect cell viability in either cell lines (Figure 6A,B). However, combination of 10 mM LiCl and 1 nM PTX had a greater inhibitory effect on cell growth at 96 h than LiCl alone (10 mM *vs*. combo, *p* = 0.02). Neither single agents nor combination treatments caused any changes in cell growth of the EM-TERT control cells.

This enhanced sensitivity to PTX was also observed in a sequential treatment regimen, in which cells were first exposed to 10 mM LiCl for 96 h followed by treatment with 1 nM PTX for another 96 h. MTT assays were performed every 24 h during the latter 96 h. 1 nM PTX had no influence on cell growth in AN3CA cells pre-exposed to control medium (Figure 6C). However, cell growth significantly slowed when they were previously exposed to 10 mM LiCl (Figure 6D, *p* = 0.00023 at 96 h). Inhibitory concentration 50 (IC_50_) was determined and pre-exposure to LiCl decreased IC_50_ of AN3CA cells to PTX from 3.59 to 1.18 nM (Figure 6E). Similarly, pre-treatment with 2 μM GSK3β inhibitor VIII for 96 h also reduced IC_50_ to PTX to 1.46 nM in AN3CA cells. It was previously reported that anti-GSK3β treatments in other cancer types, including pancreatic cancer and glioblastoma, sensitize the cells to chemotherapeutic reagents and ionizing radiation [37–40]. In particular, a recent study by Shimasaki *et al*. conducted gene profiling analyses on pancreatic cancer cell lines and revealed that GSK3β inhibition altered gene expression changes induced by chemotherapeutic drugs [39]. To determine whether GSK3β inhibition likewise changed gene regulation in endometrial cancer cells, we performed quantitative real-time RT-PCR on a set of genes related to PTX sensitization [41]. As shown in Table S1 [41,42], two of the surveyed genes, ARHGDIB and ABCB1, showed enhanced down-regulation by PTX in LiCl-sensitized AN3CA cells. These results suggest that anti-GSK3β treatment in endometrial cancer cells sensitize the cells to chemotherapeutic agent PTX.

### 2.6. LiCl Treatment Inhibits Tumor Growth *in Vivo*

Given the anti-proliferative effects seen with LiCl treatment *in vitro*, we sought to test the effects of LiCl in a murine xenograft model. First, to further characterize the cytostatic effects of LiCl treatment observed in our MTT assays, we investigated whether the G2/M accumulation was reversible. Cell cycle analyses via flow cytometry demonstrated that after 96 h of drug treatment, removal of LiCl or Inhibitor VIII for 24 h was associated with reversal of the G2/M block in viable cells (Figure S4). Given this reversibility of the drug effect on cell cycle, mice in the experimental group were given LiCl in their drinking water ad libitum to ensure a constant intake of the drug.

Previous data reported that a LiCl dose of 0.06% in the drinking water of nude mice was effective in producing clinically relevant biological and physiological responses [44,45]. We also observed an average serum lithium level of 0.9 mmol/L in J129Sv and C57BL/6 mice after 5 days of treatment with 0.06% LiCl in their drinking water (data not shown). This is comparable to the expected therapeutic range for serum lithium levels in humans (0.6–1.2 mmol/L).

**Figure 7:**
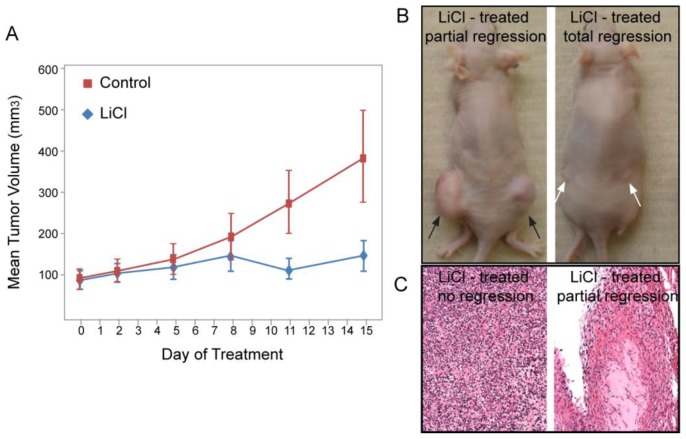
LiCl treatment reduced endometrial tumor mass *in vivo*. (**A**) Nude mice bearing AN3CA xenografts were treated with either control or LiCl-supplemented drinking water after the tumors were established (average tumor mass 100 mm^3^). Two weeks into treatment lithium-treated mice showed a significantly reduced tumor size (*p* = 0.0024); (**B**) Representative mice treated with LiCl showed partial (black arrows) or complete (white arrows) regression of the tumor; (**C**) Histological appearance of the partially regressed and fully regressed tumors.

A total of 14 mice (26 tumors) and 13 control mice (25 tumors) were treated with LiCl-water or regular drinking water, respectively. Prior to treatment start, the tumor growth rates were similar among the control and experimental arms. After 14 days of treatment, we observed a significant decrease in tumor growth rate in mice receiving LiCl compared to the control group (Figure 6A,B). Among the treated animals, there were 3 mice that had 5 tumors which completely regressed and 3 mice that had 6 tumors which partially regressed. Histologic assessment of partially regressed tumors revealed minimal residual tumor cells (Figure 7C). These results demonstrate that at a clinically approved and achievable serum level, LiCl effectively inhibit growth of endometrial cancer xenografts, which strongly suggest the potential of lithium to be used as an antineoplastic drug in the treatment of endometrial cancer.

## 3. Discussion

In this study, we investigated the effects of GSK3β inhibitors on human endometrial cancer cell growth *in vitro* and *in vivo*. We chose lithium, which as a carbonate salt is FDA-approved for the treatment of bipolar disorder, as our primary agent. Previous studies have shown that lithium acts as a reversible inhibitor of GSK3β with an IC_50_ of 2.0 mM, by both directly competing with Mg^2+^ to reduce GSK3β’s Mg^2+^:ATP-dependent kinase activity, and indirectly reducing phosphatase activity to increase the inactive form of GSK3β [46,47]. Here we showed that LiCl treatment of human endometrial cancer cell lines resulted in an elevated phospho-serine 9 level, indicating lithium inhibits GSK3β in these cells. Lithium treatment reduced endometrial cancer cell viability and proliferation by causing G2/M cell cycle arrest. Consistently, treatment with GSK3β inhibitor VIII, a highly specific and potent inhibitor of GSK3β, also resulted in similar cell cycle arrest and growth inhibition of the endometrial cancer cell lines. It is noteworthy that AN3CA and ARK1 endometrial cancer cell lines exhibited higher level of active form of GSK3β (pTyr216) compared to control EM-TERT cells, which may also contribute to their higher susceptibility to GSK3β inhibition.

The cytostatic effect of lithium on endometrial cancer cell growth retardation is achieved through induction of cell cycle arrest. Four endometrial cancer cell lines that we tested showed a significant increase in the G2/M population when exposed to lithium and a concurrent elevation in cyclin B1 level. Similar cell cycle arrest was achieved by GSK3β inhibitor VIII treatment, supporting that G2/M arrest is caused specifically by GSK3β inactivation. The molecular mechanism under this arrest, however, seems to be cell-context-dependent, as AN3CA cells showed an obvious prophase/metaphase arrest, whereas the other three cell lines appeared to be arrested at G2 phase. This pro-M phase cell cycle arrest in AN3CA cells is associated with p53 activation. On the other hand, the molecular mechanisms underlying G2 arrest in other endometrial cancer cells are unclear at present. It is possible that different genetic and epigenetic make-up of these cancer cell lines may contribute to this discrepancy.

Although at low concentration (1 mM), lithium treatment seemed to have only marginal inhibitory effect on endometrial cancer growth in cell culture, it dramatically reduced tumor growth *in vivo*. This difference between cell culture and xenografts may be attributable in part to differences in magnesium concentration present in these different systems. As mentioned above, lithium directly inhibits GSK3β kinase activity by dislodging Mg^2+^ from the Mg^2+^:ATP complex. A previous study demonstrated that reducing Mg^2+^ concentration to 0.75 mM, approximately the same free Mg^2+^ concentration found in live cells, decreases the IC_50_ of lithium from 2 to 0.8 mM, which falls well within the therapeutic range of 0.6–1.2 mmol/L [48,49]. Therefore, the high cellular concentration of Mg^2+^ in *in vitro* culture system (approximately 4 mM) would greatly dampen any effects elicited by lithium [48], and it is not unexpected that lithium would exert better GSK3β inhibition effect *in vivo* than *in vitro.* Our study using lithium chloride to target GSK3β in a murine xenograft model of endometrial cancer suggests potential efficacy as an antineoplastic. Further clinical and preclinical research is required to fully optimize the use of lithium to treat endometrial cancer.

Our observation that combined treatment of AN3CA cells with paclitaxel and lithium resulted in reduction in cell proliferation/viability greater than single agents alone further suggests potential for combined cytostatic/cytotoxic combined therapies for the treatment of endometrial cancer. Furthermore, the apparent enhanced sensitivity to PTX from pre-treating AN3CA cells with lithium chloride or GSK3β inhibitor VIII followed by low dose paclitaxel strongly supports the potential therapeutic approach for combining GSK3β inhibition and microtubule inhibitors. This sensitization was further confirmed on the molecular level, evidenced by augmented gene regulation of ARHGDIB/RhoGDI and ABCB1/MDR1 by PTX after LiCl pre-treatment. It has been long recognized the importance of combination therapies and it is well established as a superior treatment regimen over single agent chemotherapy in endometrial cancer [50,51]. Administration of multiple drugs targeting different biologic pathways may also have the benefit of reducing emergence of chemotherapy resistant cells. The timing of chemotherapy treatments in cancer patients may have a more influential role on clinical outcomes than previously appreciated. For example, circadian rhythms have been suggested to influence toxicity and drug response of combination chemotherapies in ovarian cancer [52], and abnormal circadian rhythms are associated with increased mortality in breast cancer patients [53].

This notion is further supported by our findings that lithium exposure of AN3CA endometrial cancer cells prior to paclitaxel treatment dramatically sensitized the cancer cells and significantly enhanced their responsiveness to the chemotherapeutic reagent. Further studies evaluating the combined effects of lithium and paclitaxel in additional cell lines and *in vivo* models will be required to understand whether this combined approach holds promise for the treatment of patients with recurrent or persistent endometrial cancer.

## 4. Materials and Methods

### 4.1. Cell Culture and Treatments

Endometrial cancer cell lines, AN3CA, SKUT1B, RL952, KLE, and HEC1A were obtained from American Type Culture Collection (Rockville, MD, USA). ARK1, EM-TERT [27] and ISHIKAWA were provided by Shi-Wen Jiang (Mercer University School of Medicine, Savannah, GA, USA), Pamela Pollock (Queensland University of Technology, Brisbane, Australia) and Stuart Adler (Washington University School of Medicine, St. Louis, MO, USA), respectively. HEC1A cells were maintained in DMEM:RPMI 1640 (1:1) supplemented with 10% FBS. KLE cells were maintained in DMEM:Hams F-12 (1:1) supplemented with 10% FBS. All other cells were maintained in DMEM supplemented with 10% FBS. For GSK3β inhibitor treatments, 1 M LiCl (Sigma-Aldrich, St. Louis, MO, USA) stock solution was prepared in water, sterilized and added to culture medium to final concentrations of 1 or 10 mM. 25 mM In-SolutionTM GSK3β inhibitor VIII (Calbiochem, Billerica, MA, USA) was added to culture medium to a final concentration of 10 μM.

### 4.2. MTT Assay

Endometrial cells were seeded on 96-well plates (5000 cells/well) in triplicates and treated for up to 96 h with GSK3β inhibitors. On day of assay, culture medium was replaced with 100 μL medium containing 0.83 mg/mL MTT (Sigma, St. Louis, MO, USA) and cells were incubated in MTT medium for 3 h. MTT medium was then aspirated and cells were dissolved and incubated in 150 μL MTT solvent (4 mM HCl, 0.1% NP-40 in isopropanol) for 15 min. Absorbance at 595 nm was measured on a Bio-Rad 3550 microplate reader (Bio-Rad Laboratories, Hercules, CA, USA).

### 4.3. Trypan Blue Exclusion Assay

Adherent cells as well as cells in suspension were collected by centrifugation and trypsinization, respectively. Cell pellet was resuspended in 1× PBS to 0.4~1 × 10^6^ cells/ml. 50 μL cell suspension was mixed with 50 μL 0.4% Trypan Blue solution (Sigma), and incubated at room temperature for 3 min. Viable (unstained) and nonviable (stained) cells were scored using a hemacytometer.

### 4.4. Mouse Xenograft Model

AN3CA human endometrial carcinoma cells were harvested and washed once in 1× HBSS and reconstituted in HBSS to a concentration of 2 × 10^6^/50 μL. Equal volumes of cell suspension and Matrigel (BD Biosciences, San Jose, CA, USA) were mixed and 100 μL mixture was injected subcutaneously into the dorsal flank of female NOD/SCID mice (Charles River, Wilmington, MA, USA). Width (A) and length (B) of each tumor were measured with a caliper and tumor size was calculated as A^2 × B/2. Measurements were taken every 2–3 days by two independent researchers (Yan Yin, Nora T. Kizer). Mice were sacrificed by CO_2_ inhalation after at least one tumor per mouse reached a size greater than 2 gm. Linear mixed repeated measures models were used to estimate mean tumor growth on a log scale by treatment (control/LiCl) and time and replication of experiment. Global tests for difference in mean tumor growth were performed. If the global test indicated that differences were present, linear contrasts were used to test for difference between each pair of treatments.

### 4.5. Western Blot Analyses and RT-PCR

Protein was extracted from cultured cells using lysis buffer containing a mixture of protease and phosphatase inhibitors. Equal protein loading was confirmed by reprobing membranes with a GAPDH antibody (Novus Biologicals, Littleton, CO, USA). Antibody used and dilutions are: 1:500 for anti-GSK3β (#ab73271, Abcam, Inc., Cambridge, MA, USA) and anti-phospho-GSK3β (pTyr216, sc-135653, Santa Cruz Biotechnology, Inc., Santa Cruz, CA, USA), 1:1000 for anti-phospho-GSK3β (pSer9, #TA303847, rabbit anti-human, Origene Technologies, Inc., Rockville, MD, USA), Cyclin B1 (Cell Signaling Technology, Inc., Danvers, MA, USA), total p53 (sc-126, Santa Cruz Biotechnology, Inc., Santa Cruz, CA, USA) and anti-phospho-p53 (pSer15, Cell Signaling), 1:4000 for GAPDH, 1:1250 for goat anti-rabbit IgG-HRP (sc-2030, Santa Cruz Biotechnology, Inc., Santa Cruz, CA, USA) and 1:1000 for goat anti-mouse IgG-HRP (#ab6789-1, Abcam Inc.). Phospho-Histone H3 was detected using anti-phospho-Histone H3 (Ser10, #06-570, Millipore, Temecula, CA, USA) at a dilution of 1:1000. RNA extraction and real-time RT-PCR has been described previously [54]. Sequences of primers used in this study can be found in Table S1.

### 4.6. Immunofluorescence and Confocal Microscopy

Immunofluorescence microscopy was carried out using a Zeiss Axioskop2 Plus microscope (Carl Zeiss Microscopy LLC, Thornwood, NY, USA). Images were collected using 20× objectives on an EXFO X-cite120 fluorescence illumination system using an argon laser (488 nm) and HeNe laser (633 nm). AxioVision Rel 4.5 (Carl Zeiss Microscopy) and Adobe Photoshop 7.0 (Adobe Systems, Inc., San Jose, CA, USA) were used to process the images. Nuclear staining was performed using the TO-PRO-3 iodide (642/661, # T3605, Invitrogen, Carlsbad, CA, USA). Phospho-Histone H3 was detected using anti-phospho-Histone H3 (Millipore). Alexa-Fluor 488 goat anti-rabbit IgG (#A11034, Invitrogen) was used as secondary antibody at a concentration of 1:250. Eight 200× fields were captured at random from each slide and percent positive cells for each field was calculated based off the number of phospho-Histone H3 positive cells divided by the number of total cells within each field. This was done twice by two independent reviewers and the data for each slide was combined for statistical analysis.

### 4.7. Flow Cytometry and Cell Cycle Analyses

AN3CA and EM-TERT cells were plated into 10 cm dishes at a concentration of 220,000 and 440,000 cells per dish respectively. The following day, media was removed from all dishes, and 10 mL of new media was pipetted into control plates. 10 mL of media containing either 1 mM or 10 mM LiCl was pipetted into the experimental plates. Cells were incubated for 96 h and then harvested for cell cycle analysis. Briefly cells were trypsinized, washed twice in 1× phosphate buffered saline (PBS) with 1% fetal bovine serum (FBS), and then fixed in 100% ethanol at −20°C overnight. The following day, the ethanol was removed, cells were rinsed in 1× PBS with 1% FBS and then stained with propidium iodide for analysis. Light scatter (forward, 90°) and propidium iodide signals were collected on a FacsCalibur flow cytometer (BD Biosciences). Cell cycle analyses were performed using FlowJo Software (version 7.5.5, Tree Star, Inc., Ashland, OR, USA). Nonparametric Kruskal-Wallis and Wilcoxon rank sum tests were used to compare the proportion of cells in G2/M phase by treatment (control *vs.* 1–2 doses of LiCl). If a global test indicated that differences in the median proportion were present, a similar test was used to compare all pairs of treatment groups to determine which ones had different proportions of cells in G2/M phase.

### 4.8. Lentiviral shRNA-Mediated GSK3β Knockdown

Lentiviral constructs expressing short hairpin RNAs (shRNAs) against GSK3β (shGSK3β) or Luciferase (control, shLuc) were generated in the AN3CA cell line. Virus production and infections were carried out according to established methods [55,56]. The short hairpin sequences used were: shGSK3β-1: 5′-CCAATGTTTCGTATATCTGTT-3′; shGSK3β-2: 5′-AGCAAATCAGAGAAATGAAC-3′; shLuc: 5′-CCCTCTGAACATGAGCATCAA-3′.

### 4.9. Statistical Analysis

For mitotic percentage analysis, a linear mixed model was used for each cell line to estimate mean percent of pHH3-positive cells, which is approximately Gaussian on a square root scale, by treatment (control/LiCl/GSK3β inhibitor VIII). If the global test found differences in mean percent positive cells between treatments, then linear contrasts were used to test for difference between each pair of treatments. Student’s *t*-tests were performed for the rest of the analyses if not otherwise specified, and *P*-values less than 0.05 were considered statistically significant.

## 5. Conclusions

In summary, our results support GSK3β inhibition as a potential therapeutic approach to treat endometrial cancer. In multiple human endometrial cancer cell lines, attenuating GSK3β activity by biological agents or lentiviral-mediated genetic knock-down, significantly inhibited cell growth. This cell growth inhibition is caused by a G2/M cell-cycle arrest, in a cell context-dependent manner. Treatment of AN3CA endometrial cancer cells with GSK3β inhibitors also increased sensitivity to paclitaxel, a chemotherapeutic mitotic inhibitor commonly prescribed to endometrial cancer patients. Finally, we showed in an AN3CA endometrial cancer xenograft model that GSK3β inhibition suppressed tumor growth *in vivo*.

## Supplementary Information



## References

[1] Siegel R., Ward E., Brawley O., Jemal A. (2011). Cancer statistics, 2011: The impact of eliminating socioeconomic and racial disparities on premature cancer deaths. CA: Cancer J. Clin.

[2] Temkin S.M., Fleming G. (2009). Current treatment of metastatic endometrial cancer. Cancer Control.

[3] Mizumoto Y., Kyo S., Mori N., Sakaguchi J., Ohno S., Maida Y., Hashimoto M., Takakura M., Inoue M. (2007). Activation of ERK1/2 occurs independently of KRAS or BRAF status in endometrial cancer and is associated with favorable prognosis. Cancer Sci.

[4] Pollock P.M., Gartside M.G., Dejeza L.C., Powell M.A., Mallon M.A., Davies H., Mohammadi M., Futreal P.A., Stratton M.R., Trent J.M. (2007). Frequent activating FGFR2 mutations in endometrial carcinomas parallel germline mutations associated with craniosynostosis and skeletal dysplasia syndromes. Oncogene.

[5] Byron S.A., Gartside M., Powell M.A., Wellens C.L., Gao F., Mutch D.G., Goodfellow P.J., Pollock P.M. (2012). FGFR2 point mutations in 466 endometrioid endometrial tumors: Relationship with MSI, KRAS, PIK3CA, CTNNB1 mutations and clinicopathological features. PLoS One.

[6] Dutt A., Salvesen H.B., Chen T.H., Ramos A.H., Onofrio R.C., Hatton C., Nicoletti R., Winckler W., Grewal R., Hanna M. (2008). Drug-sensitive FGFR2 mutations in endometrial carcinoma. Proc. Natl. Acad. Sci. USA.

[7] Risinger J.I., Hayes A.K., Berchuck A., Barrett J.C. (1997). PTEN/MMAC1 mutations in endometrial cancers. Cancer Res.

[8] Sun H., Enomoto T., Fujita M., Wada H., Yoshino K., Ozaki K., Nakamura T., Murata Y. (2001). Mutational analysis of the PTEN gene in endometrial carcinoma and hyperplasia. Am. J. Clin. Pathol.

[9] Stambolic V., Suzuki A., de la Pompa J.L., Brothers G.M., Mirtsos C., Sasaki T., Ruland J., Penninger J.M., Siderovski D.P., Mak T.W. (1998). Negative regulation of PKB/Akt-dependent cell survival by the tumor suppressor PTEN. Cell.

[10] Staal S.P. (1987). Molecular cloning of the akt oncogene and its human homologues AKT1 and AKT2: Amplification of AKT1 in a primary human gastric adenocarcinoma. Proc. Natl. Acad. Sci. USA.

[11] Franke T.F., Kaplan D.R., Cantley L.C. (1997). PI3K: Downstream AKTion blocks apoptosis. Cell.

[12] Rudd M.L., Price J.C., Fogoros S., Godwin A.K., Sgroi D.C., Merino M.J., Bell D.W. (2011). A unique spectrum of somatic PIK3CA (p110alpha) mutations within primary endometrial carcinomas. Clin. Cancer Res.

[13] Urick M.E., Rudd M.L., Godwin A.K., Sgroi D., Merino M., Bell D.W. (2011). PIK3R1 (p85alpha) is somatically mutated at high frequency in primary endometrial cancer. Cancer Res.

[14] Cheung L.W., Hennessy B.T., Li J., Yu S., Myers A.P., Djordjevic B., Lu Y., Stemke-Hale K., Dyer M.D., Zhang F. (2011). High frequency of PIK3R1 and PIK3R2 mutations in endometrial cancer elucidates a novel mechanism for regulation of PTEN protein stability. Cancer Discov.

[15] Velasco A., Bussaglia E., Pallares J., Dolcet X., Llobet D., Encinas M., Llecha N., Palacios J., Prat J., Matias-Guiu X. (2006). PIK3CA gene mutations in endometrial carcinoma: Correlation with PTEN and K-RAS alterations. Hum. Pathol.

[16] Shin I., Yakes F.M., Rojo F., Shin N.Y., Bakin A.V., Baselga J., Arteaga C.L. (2002). PKB/Akt mediates cell-cycle progression by phosphorylation of p27(Kip1) at threonine 157 and modulation of its cellular localization. Nat. Med.

[17] Inoki K., Li Y., Zhu T., Wu J., Guan K.L. (2002). TSC2 is phosphorylated and inhibited by Akt and suppresses mTOR signalling. Nat. Cell Biol.

[18] Cross D.A., Alessi D.R., Cohen P., Andjelkovich M., Hemmings B.A. (1995). Inhibition of glycogen synthase kinase-3 by insulin mediated by protein kinase B. Nature.

[19] Kops G.J., Dansen T.B., Polderman P.E., Saarloos I., Wirtz K.W., Coffer P.J., Huang T.T., Bos J.L., Medema R.H., Burgering B.M. (2002). Forkhead transcription factor FOXO3a protects quiescent cells from oxidative stress. Nature.

[20] Shakoori A., Mai W., Miyashita K., Yasumoto K., Takahashi Y., Ooi A., Kawakami K., Minamoto T. (2007). Inhibition of GSK-3 beta activity attenuates proliferation of human colon cancer cells in rodents. Cancer Sci.

[21] Mussmann R., Geese M., Harder F., Kegel S., Andag U., Lomow A., Burk U., Onichtchouk D., Dohrmann C., Austen M. (2007). Inhibition of GSK3 promotes replication and survival of pancreatic beta cells. J. Biol. Chem.

[22] Cao Q., Lu X., Feng Y.J. (2006). Glycogen synthase kinase-3beta positively regulates the proliferation of human ovarian cancer cells. Cell Res.

[23] Kang T., Wei Y., Honaker Y., Yamaguchi H., Appella E., Hung M.C., Piwnica-Worms H. (2008). GSK-3 beta targets Cdc25A for ubiquitin-mediated proteolysis, and GSK-3 beta inactivation correlates with Cdc25A overproduction in human cancers. Cancer Cell.

[24] Miyashita K., Nakada M., Shakoori A., Ishigaki Y., Shimasaki T., Motoo Y., Kawakami K., Minamoto T. (2009). An emerging strategy for cancer treatment targeting aberrant glycogen synthase kinase 3 beta. Anti-Cancer Ag. Med. Chem.

[25] Wang Z., Smith K.S., Murphy M., Piloto O., Somervaille T.C., Cleary M.L. (2008). Glycogen synthase kinase 3 in MLL leukaemia maintenance and targeted therapy. Nature.

[26] Gustin J.P., Karakas B., Weiss M.B., Abukhdeir A.M., Lauring J., Garay J.P., Cosgrove D., Tamaki A., Konishi H., Konishi Y. (2009). Knockin of mutant PIK3CA activates multiple oncogenic pathways. Proc. Natl. Acad. Sci. USA.

[27] Mizumoto Y., Kyo S., Ohno S., Hashimoto M., Nakamura M., Maida Y., Sakaguchi J., Takakura M., Inoue M., Kiyono T. (2006). Creation of tumorigenic human endometrial epithelial cells with intact chromosomes by introducing defined genetic elements. Oncogene.

[28] Yazlovitskaya E.M., Edwards E., Thotala D., Fu A., Osusky K.L., Whetsell W.O., Boone B., Shinohara E.T., Hallahan D.E. (2006). Lithium treatment prevents neurocognitive deficit resulting from cranial irradiation. Cancer Res..

[29] Li X., Friedman A.B., Zhu W., Wang L., Boswell S., May R.S., Davis L.L., Jope R.S. (2007). Lithium regulates glycogen synthase kinase-3beta in human peripheral blood mononuclear cells: Implication in the treatment of bipolar disorder. Biol. Psychiatry.

[30] Bhat R., Xue Y., Berg S., Hellberg S., Ormo M., Nilsson Y., Radesater A.C., Jerning E., Markgren P.O., Borgegard T. (2003). Structural insights and biological effects of glycogen synthase kinase 3-specific inhibitor AR-A014418. J. Biol. Chem..

[31] Wakefield J.G., Stephens D.J., Tavare J.M. (2003). A role for glycogen synthase kinase-3 in mitotic spindle dynamics and chromosome alignment. J. Cell Sci.

[32] Yang K., Guo Y., Stacey W.C., Harwalkar J., Fretthold J., Hitomi M., Stacey D.W. (2006). Glycogen synthase kinase 3 has a limited role in cell cycle regulation of cyclin D1 levels. BMC Cell Biol.

[33] Diehl J.A., Cheng M., Roussel M.F., Sherr C.J. (1998). Glycogen synthase kinase-3beta regulates cyclin D1 proteolysis and subcellular localization. Genes Dev.

[34] Kulikov R., Boehme K.A., Blattner C. (2005). Glycogen synthase kinase 3-dependent phosphorylation of Mdm2 regulates p53 abundance. Mol. Cell. Biol.

[35] Fang F., Newport J.W. (1991). Evidence that the G1-S and G2-M transitions are controlled by different cdc2 proteins in higher eukaryotes. Cell.

[36] Levine A.J. (1997). p53, the cellular gatekeeper for growth and division. Cell.

[37] Miyashita K., Kawakami K., Nakada M., Mai W., Shakoori A., Fujisawa H., Hayashi Y., Hamada J., Minamoto T. (2009). Potential therapeutic effect of glycogen synthase kinase 3beta inhibition against human glioblastoma. Clin. Cancer Res.

[38] Mamaghani S., Patel S., Hedley D.W. (2009). Glycogen synthase kinase-3 inhibition disrupts nuclear factor-kappaB activity in pancreatic cancer, but fails to sensitize to gemcitabine chemotherapy. BMC Cancer.

[39] Shimasaki T., Ishigaki Y., Nakamura Y., Takata T., Nakaya N., Nakajima H., Sato I., Zhao X., Kitano A., Kawakami K. (2012). Glycogen synthase kinase 3beta inhibition sensitizes pancreatic cancer cells to gemcitabine. J. Gastroenterol.

[40] Kitano A., Shimasaki T., Chikano Y., Nakada M., Hirose M., Higashi T., Ishigaki Y., Endo Y., Takino T., Sato H. (2013). Aberrant glycogen synthase kinase 3beta is involved in pancreatic cancer cell invasion and resistance to therapy. PLoS One.

[41] Goto T., Takano M., Sakamoto M., Kondo A., Hirata J., Kita T., Tsuda H., Tenjin Y., Kikuchi Y. (2006). Gene expression profiles with cDNA microarray reveal RhoGDI as a predictive marker for paclitaxel resistance in ovarian cancers. Oncol. Rep.

[42] Spandidos A., Wang X., Wang H., Seed B. (2010). PrimerBank: a resource of human and mouse PCR primer pairs for gene expression detection and quantification. Nucleic Acids Res.

[43] *ED50plus*, version1.0.

[44] Roybal K., Theobold D., Graham A., DiNieri J.A., Russo S.J., Krishnan V., Chakravarty S., Peevey J., Oehrlein N., Birnbaum S. (2007). Mania-like behavior induced by disruption of CLOCK. Proc. Natl. Acad. Sci. USA.

[45] Polotsky A.J., Zhu L., Santoro N., Pollard J.W. (2009). Lithium chloride treatment induces epithelial cell proliferation in xenografted human endometrium. Hum. Reprod.

[46] Klein P.S., Melton D.A. (1996). A molecular mechanism for the effect of lithium on development. Proc. Natl. Acad. Sci. USA.

[47] Jope R.S. (2003). Lithium and GSK-3: One inhibitor, two inhibitory actions, multiple outcomes. Trends Pharmacol. Sci.

[48] Ryves W.J., Harwood A.J. (2001). Lithium inhibits glycogen synthase kinase-3 by competition for magnesium. Biochem. Biophys. Res. Commun.

[49] Srisurapanont M., Pratoomsri W., Maneeton N. (2000). Evaluation of three simple methods for predicting therapeutic lithium doses. Psychiatry Res.

[50] Fleming G.F., Filiaci V.L., Bentley R.C., Herzog T., Sorosky J., Vaccarello L., Gallion H. (2004). Phase III randomized trial of doxorubicin + cisplatin *versus* doxorubicin + 24-h paclitaxel + filgrastim in endometrial carcinoma: A Gynecologic Oncology Group study. ESMO.

[51] Thigpen J.T., Brady M.F., Homesley H.D., Malfetano J., DuBeshter B., Burger R.A., Liao S. (2004). Phase III trial of doxorubicin with or without cisplatin in advanced endometrial carcinoma: A gynecologic oncology group study. J. Clin. Oncol.

[52] Hrushesky W.J. (1985). Circadian timing of cancer chemotherapy. Science.

[53] Sephton S.E., Sapolsky R.M., Kraemer H.C., Spiegel D. (2000). Diurnal cortisol rhythm as a predictor of breast cancer survival. J. Natl. Cancer Inst.

[54] Lin C., Yin Y., Bell S.M., Veith G.M., Chen H., Huh S.H., Ornitz D.M., Ma L. (2013). Delineating a conserved genetic cassette promoting outgrowth of body appendages. PLoS Genet.

[55] Stewart S.A., Dykxhoorn D.M., Palliser D., Mizuno H., Yu E.Y., An D.S., Sabatini D.M., Chen I.S., Hahn W.C., Sharp P.A. (2003). Lentivirus-delivered stable gene silencing by RNAi in primary cells. RNA.

[56] Chiappinelli K.B., Haynes B.C., Brent M.R., Goodfellow P.J. (2012). Reduced DICER1 elicits an interferon response in endometrial cancer cells. Molecular Cancer Res.

