# Supplementary material for: Glycogen Synthase Kinase 3β Inhibition as a Therapeutic Approach in the Treatment of Endometrial Cancer
Source: Int J Mol Sci. 2013 Aug 12;14(8):16617–37. doi: 10.3390/ijms140816617 (PMC3759928; doi:10.3390/ijms140816617)
Supplement: Supplementary file 1 [file ijms-14-16617-s001.pdf]

Supplementary Information

Figure S1. LiCl treatment induces GSK3β inhibition in endometrial cancer cells. (**A**) Western blot analysis of total GSK3β and pSer9 GSK3β for EM-TERT and AN3CA after indicated hours of treatment with 10mM LiCl. Increased phosphorylation at serine 9 residue of GSK3β (pSer9 GSK3β) observed in both cell lines as early as 24 h; (**B**) Normalization of pSer9 to total GSK3 showing that the relative increase in pSer9 GSK3β is more pronounced in AN3CA cells and that the ratio of pSer9 GSK3β to total GSK3β increases overtime.

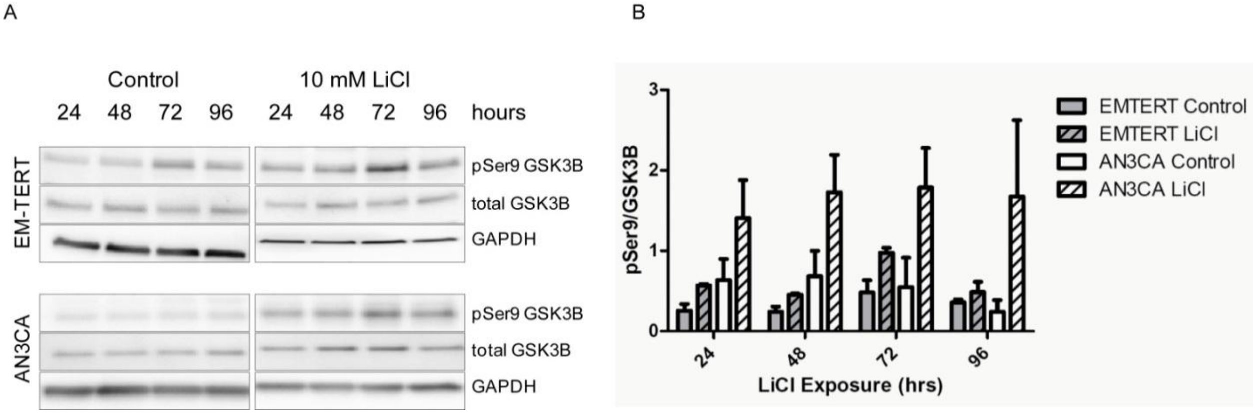

Figure S2. Trypan Blue exclusion test on endometrial cell lines treated with no drug (control), LiCl or GSK3 inhibitor VIII. At 10 mM, LiCl treatment increased cell death in AN3CA and ARK1 cells, without affecting EM-TERT cells. GSK3 inhibitor VIII on the other hand exhibited varied cell toxicity in all three cell lines with greatest fold increases in nonviable cells seen in the AN3CA cells.

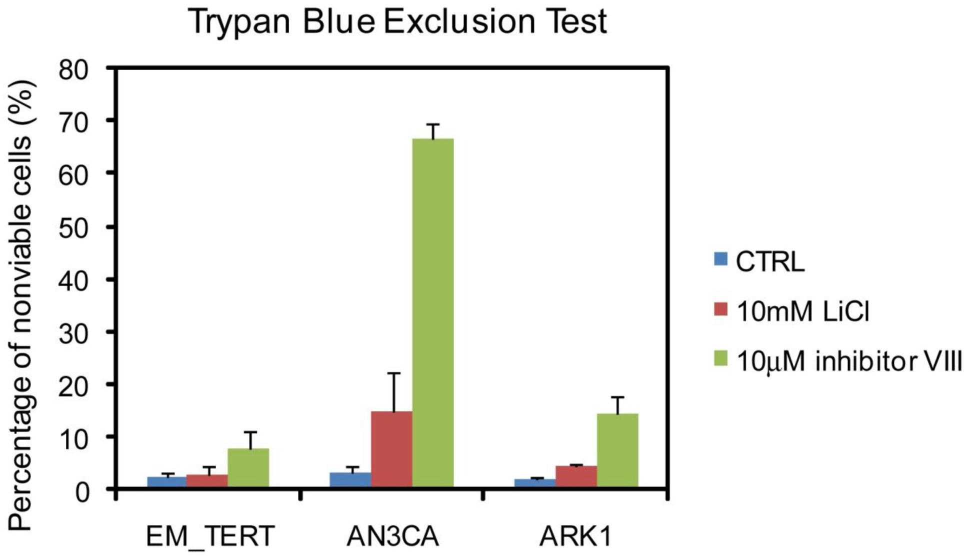

Figure S3. Representative FACS analyses on cell cycle progression in untreated (red) and 10 μM GSK3β inhibitor VIII-treated (blue) endometrial cell lines. In AN3CA cells, percentage of cells in G2/M phase steadily increased and peaked at 96 h. No such changes were detected in the EM-TERT cell line. Percentages of cells in G2/M are indicated.

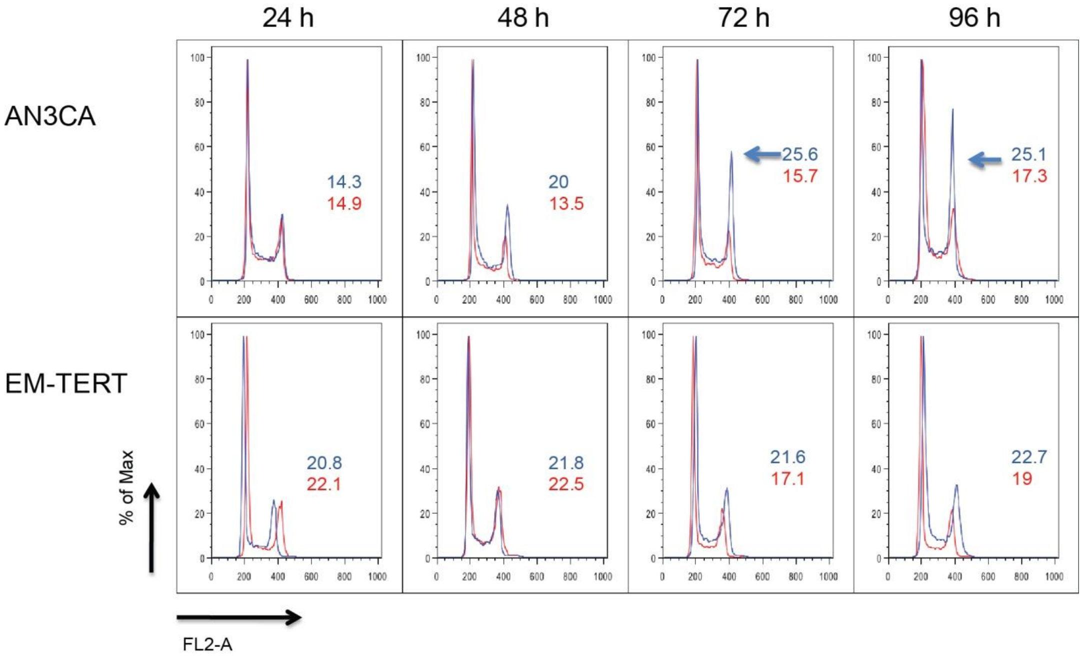

Table S1. Gene expression changes in AN3CA cells treated with PTX alone or in combination with LiCl.

Gene name	Accession number	PCR primers sequence	Fold-change PTX (*p*-value compared to control)	Fold-change PTX + LiCl (*p*-value compared to PTX alone)
*ARHGDIB	NM_001175	5'GACTGGGGTGAAAGTGGATAAAG3'<br>5'TCGTCGGTGAAGAAGGACTTG3'	0.52 (0.012)	0.30 (0.035)
*ABCB1	NM_000927	5'TTGCTGCTTACATTCAGGTTTCA3'<br>5'AGCCTATCTCCTGTCGCATTA3'	0.78 (0.039)	0.46 (0.005)
KRT4	NM_002272	5'CGCGAACAGATCAAGCTCCT3'<br>5'GGGGCTCAAGGTTTTTGCTG3'	0.69 (0.048)	0.52 (0.30)
PDAP1	NM_014891	5'CAAAGGAGCTTTCGAGGAGAG3'<br>5'GTGGCATCGTCTTTTGCTTTC3'	1.23 (0.29)	1.19 (0.78)
FDFT1	NM_004462	5'ACTTCCCAACGATCTCCCTTG3'<br>5'CCCATTCTCCGGCAAATGTC3'	0.89 (0.45)	0.81 (0.24)
IFITM2	NM_00643	5'ATGAACCACATTGTGCAAACCT3'<br>5'CCCAGCATAGCCACTTCCT3'	0.66 (0.031)	0.62 (0.32)
TRIP6	NM_003302	5'GTGGGCTGCTTTGTATGTTCT3'<br>5'GCCCTCGCAATATGCCCTC3'	1.15 (0.47)	0.98 (0.62)

The list of genes are based on a previous study [41]; Primer sequences were obtained from PrimerBank database [42];
* Genes showed augmented changes by PTX in LiCl-treated cells.

Figure S4. Rapid return to pre-treatment cell cycle phases upon removal of LiCl. 24 h after LiCl-withdrawal, the LiCl-induced G2/M arrest started to resolve.

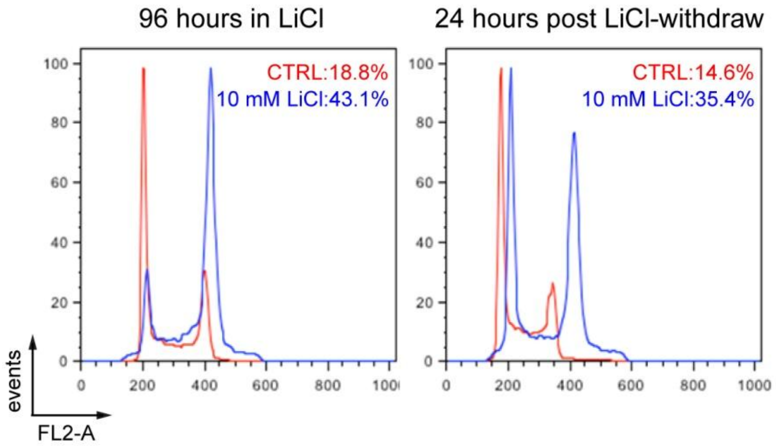

© 2013 by the authors; licensee MDPI, Basel, Switzerland. This article is an open access article distributed under the terms and conditions of the Creative Commons Attribution license (http://creativecommons.org/licenses/by/3.0/).
